# Comparison of Three Different Chemotherapy Regimens Containing Epirubicin in Hormone-Refractory Prostate Cancer Patients

**DOI:** 10.1100/tsw.2008.90

**Published:** 2008-06-13

**Authors:** Hamit Ersoy, Orhan Yigitbası, Levent Sagnak, Hikmet Topaloglu, Ahmet Kiper

**Affiliations:** ^1^ Ankara Diskapi Education and Research Hospital, 3^rd^ Urology Clinic, Ankara, Turkey; ^2^ Ankara Diskapi Education and Research Hospital, ^1st^ Urology Clinic, Ankara, Turkey; ^3^ Mustafa Kemal University, Department of Urology, Hatay, Turkey

**Keywords:** hormone-refractory prostate cancer, epirubicin, estramustine phosphate

## Abstract

We compared three different chemotherapy regimens containing epirubicin in hormonerefractory prostate cancer (HRPC) patients. Sixty-nine patients with HRPC were randomized into three groups. The first group (22 patients) received 30 mg/m^2^/week i.v. epirubicin for 8 weeks. The second group (24 patients) received 30 mg/m^2^/week i.v. epirubicin for 8 weeks followed by monthly maintenance therapy for 4–6 months. The third group (23 patients) received oral estramustine phosphate (EMP) at a dose of 840 mg/day together with weekly and monthly maintenance epirubicin. The response rates, mean survival times, and toxicity were determined. Within the first 3 months, pain and performance scores were improved by at least one degree in all the groups. One patient in group two and three patients in group three had complete response. Partial response rates were 23% in group 1, 25% in group 2, and 17% in group 3. Stable disease rates were 41% in group 1, 33% in group 2, and 26% in group 3. The progression rates within the first 3 months were 36% in group 1, 38% in group 2, and 44% in group 3. None of the patients developed complications that were significant enough to terminate the treatment. Two patients in group 3 died of cardiotoxicity. The mean survival times were 10.1, 15.8, and 16.1 months in groups 1, 2, and 3, respectively. It was determined that weekly and maintenance epirubicin treatment protocol, and estramustine treatment protocol in addition to this treatment, was only meaningfully more effective against weekly epirubicin treatment in the statistical sense (0.01 < p < 0.05). However, due to the complications of EMP, which influence the quality of life, we believe that this was usable only when measures were adopted against these effects.

## INTRODUCTION

Despite the fact that several cytostatic agents are used in the treatment of hormone-refractory prostate cancer (HRPC), these medications have only palliative effects and there are no established standard treatment protocols. Their use either alone or in combination with other drugs has not been shown to have any significant effect on life expectancy as well[1,2]. In the utilization of either single or combined drug regimens, in addition to subjective response, objective response rates of 6.5% and disease stability rates of 15% have also been reported[3]. However, to date, combination therapy does not appear to be superior to single agents[4].

One of the most commonly used medications for HRPC patients is epirubicin. Epirubicin is an anthracycline derivative that exerts its cytostatic effects by binding to DNA and creating irreversible helix breaks. Compared to doxorubicin, epirubicin has better antitumor activity and is associated with less toxicity[5]. There is no established standard protocol for epirubicin. In the literature, weekly low-dosage schemes (12–35 mg/m$^2$), as well as high-dosage schemes (60–100 mg/m$^2$), every 3–4 weeks have been reported[6,7]. In addition to having myelosuppressive effects, epirubicin has myocardial toxicity that is observed in cumulative doses exceeding 900 mg/m$^2$[5].

In patients with HRPC, estramustine phosphate (EMP) is used in most of the combined chemotherapy regimens. It is synthesized by combining estradiol with nitrogen mustard, which allows selective delivery of the alkylating agent into estrogen receptor–positive tumor cells[8]. EMP shows its antimitotic effects by binding to tubulin-binding domains of microtubule-related proteins[9]. In recent trials, the combination of EMP with vinblastine or etoposide has given promising results in the treatment of HRPC[10].

In this study, we determined the effects of epirubicin in three different regimens combined with EMP on survival, response rate, toxicity, and quality of life in patients with HRPC.

## MATERIALS AND METHODS

Sixty-nine patients with advanced prostate cancer who had progressed during hormonal therapy were recruited into the study. Patients receiving luteinizing hormone-releasing hormone analogues continued their treatment during chemotherapy to maintain the serum testosterone at castration levels. The exclusion criteria were previous history of myocardial infarction, congestive heart failure, angina, arrhythmia, and previous chemotherapy. Pretreatment evaluations included medical history, physical examination, cardiovascular examination, performance status (able to carry out all normal activity without restriction = 0; restricted in physically strenuous activity, but ambulatory and able to do light work = 1; ambulatory and capable of self-care, but unable to carry out any work = 2; capable of only limited self-care confined to bed or chair 50% or more of waking hours = 3; completely disabled and cannot carry out any self-care = 4), pain score (analgesics not required = 0, non-narcotics occasionally required = 1, non-narcotics regularly required = 2, narcotics occasionally required = 3, narcotics regularly required = 4), complete blood count, routine biochemical profile (alkaline phosphatase, serum electrolytes, blood urea nitrogen, creatinine, glucose, calcium, total bilirubin, aspartate amonitransferase), and ECG. Imaging studies included chest X-ray, abdominal ultrasonography, abdominopelvic computed tomography (CT), and radionuclide bone scan. The extent of disease (EOD) on bone scan was determined according to the criteria of Soloway et al.[11]. Patients with a pain score and performance score above 2, according to the World Health Organization (WHO) criteria, were excluded from the study. Serum prostate specific antigen (PSA) levels and testosterone levels were also evaluated prior to treatment. Clinical characteristics of patients are summarized in Table 1.

The patients were randomized into three groups of treatment. Twenty-two patients in group 1 received i.v. epirubicin at a dose of 30 mg/m$^2$ once a week for 8 weeks, while 24 patients in group 2 received i.v. epirubicin at a dose of 30 mg/m$^2$ once a week for 8 weeks followed by 30 mg/m$^2$ i.v. epirubicin once a month for 4–6 months. Twenty-three patients in group 3 were given the same treatment as patients in group 2 in addition to oral EMP three times daily 2 h after meals, for a total daily dose of 840 mg. All the patients were informed about the adverse effects and had given consent before the study.

**TABLE 1**
**Patient Characteristics**

| | Group 1 | Group 2 | Group 3 |
|---|---|---|---|
| No. of patients | 22 | 24 | 23 |
| Age (year) | 67[a] (52–81)[b] | 65[a] (54–75)[b] | 66[a] (49–79)[b] |
| Serum PSA (ng/dl) | 81[a] (25–190)[b] | 96[a] (11–287)[b] | 110[a] (9–520)[b] |
| Prior therapy | | | |
| Orchiectomy + flutamide | 4 | 16 | — |
| Orchiectomy + CPA | 18 | 8 | — |
| Orchiectomy + bicalutamide | — | — | 13 |
| LHRHa + bicalutamide | — | — | 10 |
| Progression time (months) | 18[a] (4–36)[b] | 24[a] (6–72)[b] | 17[a] (6–40)[b] |
| Measurable soft tissue lesions | | | |
| Lymph nodes | 2 | 5 | 4 |
| Liver | 3 | 1 | 2 |
| Local recurrence (urinary obstruction) | 5 | 3 | 3 |
| Skeletal metastases, EOD grading | | | |
| 0 | — | — | — |
| 1 | 8 | 8 | 6 |
| 2 | 9 | 11 | 14 |
| 3 | 4 | 5 | 3 |
| 4 | 1 | — | — |
| WHO performance status | | | |
| 0 | 15 | 14 | 13 |
| 1 | 4 | 7 | 8 |
| 2 | 3 | 3 | 2 |
| Pain score | | | |
| 0 | 3 | 11 | 2 |
| 1 | 12 | 7 | 14 |
| 2 | 7 | 7 | 7 |
| Mean pain score | 1.2 | 0.84 | 1.27 |
| Mean performance status | 0.45 | 0.52 | 0.52 |
| Mean PSA (pretreatment) | 81 (25–190) | 96 (11–287) | 110 (9–520) |

PSA, prostate-specific antigen; CPA, cyproterone acetate; LHRHa, luteinizing hormone-releasing hormone analog; [a]Median, [b]Range.

Evaluation during the study included complete blood count every 2 weeks, biochemical profile and serum PSA levels every 4 weeks, abdominal ultrasonography every 2 months, and CT when deemed necessary. Radionuclide bone scan was repeated every 6 months. Pain and performance scores of the patients were evaluated monthly. For the assessment of toxicity, the WHO toxicity grading system was used[12]. All the patients were followed up for 4–36 months or until they died.

All the patients were evaluated for response according to National Prostatic Cancer Treatment Group (NPCTG) response criteria[13]. A complete response (CR) was defined as normalization of the PSA level and in patients with measurable disease, disappearance of all lesions without the occurrence of new ones for at least 4 weeks. A partial response (PR) was defined as a decrease of ≥50% in the sum of the products of the longest diameters of all measurable lesions persisting for ≥4 weeks, improvement in bone scan findings, and reossification of lytic lesions, in addition to no increase in the size of any existing lesions, no appearance of new lesions, and a decrease of at least 50% or more in the PSA baseline value on three measurements, obtained 3 weeks apart. Stable disease (SD) was defined as a decrease of <50% or an increase in tumor size of <25% as compared with the original measurements, and a decrease of <50% or an increase of <25% of the PSA baseline values on three measurements, obtained 3 weeks apart. Progressive disease (PD) was defined as any increase of ≥25% in the sum of the products of the longest diameters of any measurable lesions or the appearance of new lesions, and an increase of 50% or greater in PSA levels from the baseline value. Survival duration was measured from the start of the treatment to the date of death or last follow-up.

## Statistical Analysis

Statistical analysis was performed by Statistical Package for Social Sciences (SPSS) 11.5 software (SPSS Inc., Chicago, IL). Whether the continuous variables were normally distributed or not were determined by using the Shapiro Wilk test. Whereas continuous data were expressed as mean (minimum-maximum), otherwise number of patients and (%) were used for categorical data. Kruskal Wallis variance analysis was applied for determining the differences among groups regarding for continuous and ordinal variables. When the *p*-value from the Kruskal-Wallis test statistics was statistically significant, a multiple comparison test was used to know which groups differed from which others. The differences among repeated pain and PSA measures were evaluated by using the Friedman test. When the *p*-value from the Friedman test statistics was statistically significant, the Wilcoxon Sign Rank test was used to know which measurement time differed from which others. The Kaplan-Meier method was used to analyze survival and comparisons of survival curves between treatment groups were made with the log-rank test. A *p*-value <0.05 was considered statistically significant.

## RESULTS

At the end of the first 3 months, other than one patient in group 2, the pain and performance scores of the other patients in the three groups improved by at least one grade. Despite these improvements in the subjective findings, the same improvement was not observed in the objective findings (Table 2).

**TABLE 2**
**Response to Treatment with Respect to the Group at the End of 3 Months, Starting from the Commencement of Treatment**

| Clinical Response | Group 1 (22 Patients) Patient No.; % | Group 2 (24 Patients) Patient No.; % | Group 3 (23 Patients) Patient No.; % |
|---|---|---|---|
| Complete response | — | 1; 4% | 3; 13% |
| Partial response | 5; 23% | 6; 25% | 4; 17% |
| Stable | 9; 41% | 8; 33% | 6; 26% |
| Progression | 8; 36% | 9; 38% | 10; 44% |

In the first group, tomographical remission was observed in three patients with liver metastasis. However, no remission was seen in the liver metastasis observed in the three patients in the other two groups. Also, in all three groups, there was edema in the feet in connection with the lymphadenopathy, determined with ultrasound and tomography. This edema started to improve in all patients after the 2$^{nd}$

and 3rd months. However, the edema increased in all patients, starting from the 6th month, to the extent that walking became difficult. Additional corticosteroids were administered to two patients in groups 1 and 2 who had neurological complaints in connection with medulla spinalis pressure and who could not walk. Until the end of the 2nd month, the patients were able to walk with assistance. Percutaneous nephrostomy was performed in two of the five patients with urethral obstruction in group 1 and in all of the three patients in other groups. Transition to bladder was observed in pyelography in only one patient in group 2 and nephrostomy was taken.

PSA value, assessed as an objective finding, decreased by more than 50% in five patients in group 1. Three of these were patients with liver metastasis. These patients were accepted as partial remission. Nine patients in this group were accepted as stable and eight patients as progression. Pain score, which averaged 1.2, decreased to 0.3 and performance score from 0.45 to 0.15. Average PSA value decreased to 67 (8–60).

In the second group, PSA descended to the normal value and grade 1 bone metastasis disappeared. This patient was accepted as complete remission. Partial remission was observed in six patients and stable condition in eight patients. Although nine patients were accepted as progression with objective findings, improvements were seen in pain and performance scores of other patients, other than one patient. The average pain score, which was 0.84 before the treatment, decreased to 0.35 and performance score decreased from 0.52 to 0.12. In this group, it was observed that the average PSA value decreased to 41 (3–166).

In three patients in the third group, PSA descended to the normal value and grade 1 and 2 bone metastases disappeared. Partial remission was observed in four patients and stable condition in six patients. Although progression was observed in 10 patients, subjective findings improved in all patients. The average pain score, which was 1.27 before the treatment, decreased to 0.22 and performance score decreased from 0.52 to 0.10. Similarly, average PSA value decreased to 48 (1–190) (Table 2, Fig. 1.)

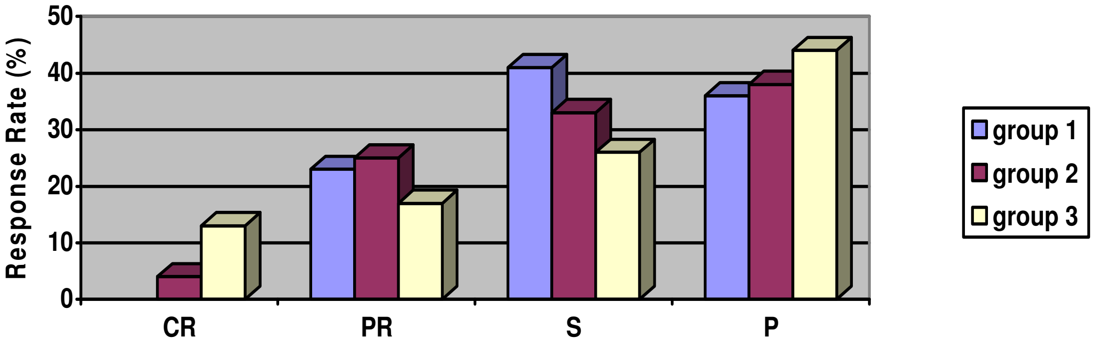

**FIGURE 1.** The assessment of response according to the treatment groups at the end of 3 months (CR: complete response, PR: partial response, S: Stable, P: progression).

When we evaluated the variations in pain score statistically in all of the three groups at the 3rd month of treatment, statistically significant differences were measured between the groups ($p$ = 0.002). According to this, the improvement in the pain scores of group 1 and 3 was more significant vs. group 2 ($p$ = 0.024 and $p$ < 0.001). There was no significant difference between groups 1 and 3 ($p$ = 0.301). In the same way, a statistically significant difference was observed in post-treatment PSA levels between the groups ($p$ < 0.001). According to this, the decrease in PSA levels between groups 2 and 3 vs. group 1 was more significant statistically ($p$ < 0.001 and $p$ < 0.001). There was no significant difference between groups 2 and 3 ($p$ = 0.773).

At the end of 6 months, progression was observed in 16 of the 22 patients in group 1 (73%). Similarly, the average pain score increased to 0.72 and performance score to 0.4. PSA average was 96

(44–180). In 11 of the 23 patients who survived (48%), progression was observed. In this group, it was observed that average pain score increased to 0.6 and performance score to 0.35. In this group, it was seen that the PSA average was 48 (22–170).

In the third group, progression was observed only in seven of the 22 patients who survived (32%). The average pain score in this group was observed as 0.3 and performance score as 0.3. PSA average decreased to 36 (2–160) (Table 3, Fig. 2).

**TABLE 3**
**Response to Treatment at the End of 6th Month**

| Clinical Response | Group 1 (22 Patients) Patient No.; % | Group 2 (23 Patients) Patient No.; % | Group 3 (22 Patients) Patient No.; % |
|---|---|---|---|
| Complete response | — | — | 2; 9% |
| Partial response | — | 4; 17% | 5; 23% |
| Stable | 6; 27% | 8; 34% | 8; 36% |
| Progression | 16; 73% | 11; 49% | 7; 32% |

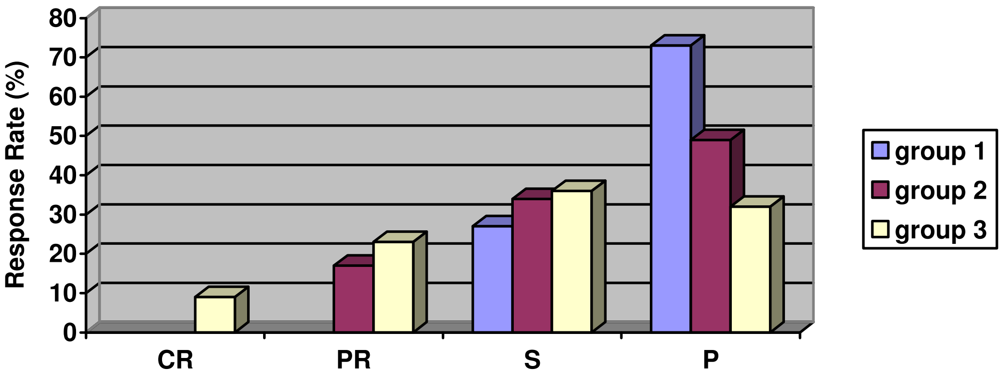

**FIGURE 2**. The assessment of response according to the treatment groups at the end of 6 months (CR: complete response, PR: partial response, S: Stable, P: progression).

The differences in pain score at the 6-month follow-up were statistically significant in all of the three groups ($p < 0.001$). According to this, the improvement in pain score was more significant in group 3 vs. groups 1 and 2 ($p = 0.002$ and $p < 0.001$). There was no significant difference between groups 1 and 2 ($p = 0.061$). It was also evaluated that the differences in PSA measurements were statistically significant in this 6-month follow-up in all groups ($p < 0.001$). There was a decrease in PSA levels of groups 2 and 3, whereas an increase was seen in group 1. The improvement was more significant statistically in groups 2 and 3 vs. group 1 ($p < 0.001$ and $p < 0.001$). There was no significant difference between groups 2 and 3 ($p = 0.522$).

At the 9-month follow-up of the patients, progression had developed in 85% of 18 patients who survived in group 1. The average pain score in this group was observed as 1.3 and performance score as 0.75. It was observed that PSA average increased to 106 (88–190). While progression was observed in 60% of the 20 patients who survived in group 2, pain score was 0.94 and performance score was 0.7. Also, average PSA value decreased to 72 (35–190). In the third group, progression was observed in 55% of the 20 patients who survived. The average pain score in this group was observed as 0.6 and performance score as 0.6. PSA average decreased to 58 (4–185) (Table 4, Fig. 3).

**TABLE 4**
**Response to Treatment at the End of 9th Month**

| Clinical Response | Group 1 (18 Patients) Patient No.; % | Group 2 (20 Patients) Patient No.; % | Group 3 (20 Patients) Patient No.; % |
|---|---|---|---|
| Complete response | — | — | 1; 5% |
| Partial response | — | 3; 15% | 4; 20% |
| Stable | 3; 16% | 5; 25% | 4; 20% |
| Progression | 15; 84% | 12; 60% | 11; 55% |

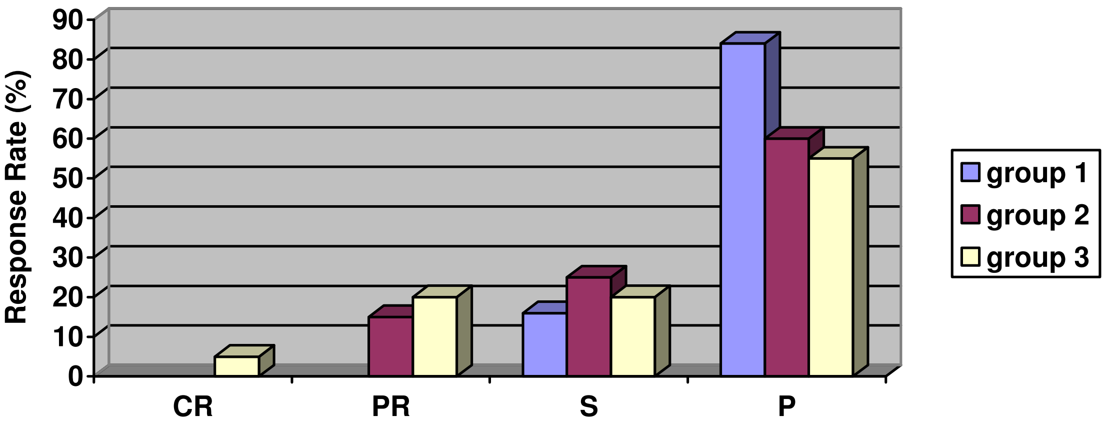

**FIGURE 3**. Response to treatment at the end of the 9th month with respect to treatment groups.

At the 9-month follow-up of the patients, the differences in pain score between the groups were statistically significant ($p < 0.001$). According to this, as pain levels decreased in group 3, a statistically significant increase was observed in groups 1 and 2 ($p < 0.001$ and $p < 0.001$). There was no significant difference between groups 1 and 2 ($p = 0.690$). According to PSA levels, there also were statistically significant differences between the three groups ($p < 0.001$). The PSA levels decreased in groups 2 and 3, and there was an increase in group 1 that was statistically significant ($p < 0.001$ and $p < 0.001$). There was no significant difference between groups 2 and 3 ($p = 0.6$).

The graphics concerning the pain and performance scores of the patients at the follow-up of the initial 9 months are given in Figs. 4 and 5, and the average PSA values of the patients in the first 9 months are given in Fig. 6.

Every treatment group was evaluated for pain and PSA levels. In group 1 patients, an increase was observed from the 4th month of follow-up in subjective and objective findings, and according to time, there was a statistically significant difference in pain and PSA levels ($p < 0.001$). The pain score showed a statistically significant decrease at the 3rd and 6th months ($p < 0.001$ and $p = 0.002$), whereas there was a significant increase at the 9th month ($p = 0.025$). PSA levels showed a significant decrease at the 3rd month ($p < 0.001$), nonsignificant increase at the 6th month ($p = 0.179$), and a significant increase at the 9th month ($p = 0.008$).

In group 2, a progression was observed from the 6th month of follow-up and according to time, a statistically significant difference was observed in pain and PSA levels ($p < 0.001$). The pain score was significantly decreased at the 3rd and 6th months ($p = 0.003$ and $p = 0.046$), when an increase was observed at the 9th month ($p = 0.008$). A statistically significant decrease was observed in PSA levels at the 3rd and 6th months of follow-up that was not significant at the 9th month ($p = 0.024$).

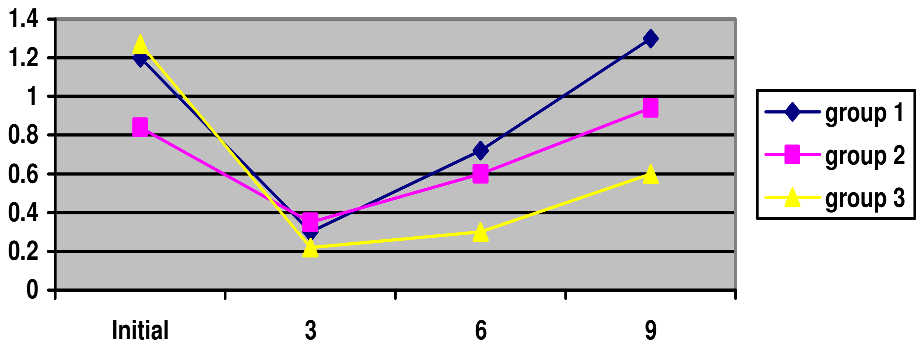

**FIGURE 4**. The change of pain scores of patients in three groups in time (months).

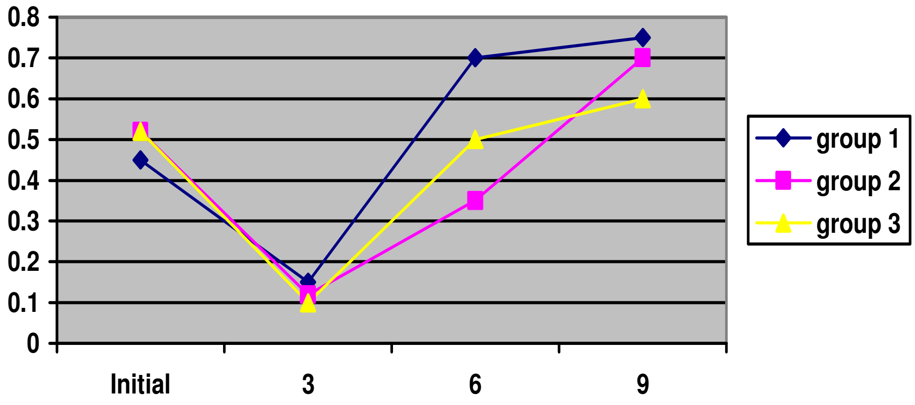

**FIGURE 5**. The change of performance scores of patients in three groups in time (months).

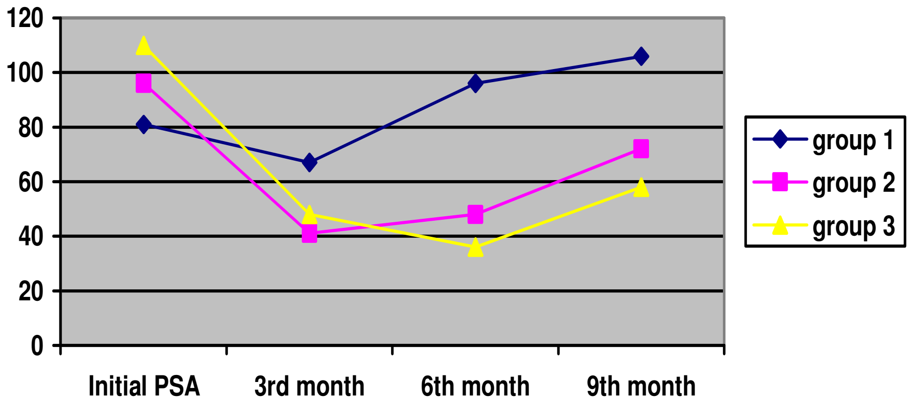

**FIGURE 6.** The PSA average change graphics of the patients, before and after the treatment.

In group 3 patients, progression was observed after 8.4 months and according to time, there was a significant difference in pain and PSA levels ($p < 0.001$). In this group, the pain score showed a statistically significant decrease at the 3$^{rd}$, 6$^{th}$, and 9$^{th}$ months of follow-up ($p < 0.001$, $p < 0.001$, and $p = 0.003$). However, PSA levels showed a significant decrease at the 3$^{rd}$ and 6$^{th}$ months ($p < 0.001$ and $p < 0.001$), but a nonsignificant decrease at the 9$^{th}$ month ($p = 0.061$).

When the patients were assessed with respect to their life spans, the average life span of the patients in the first group was seen as 10.3 months, 15.8 months in the second group, and 16.5 months in the third group ($0.01 < p < 0.05$)(Fig. 7). Consequently, it was observed that with respect to treatment response time and life span, monthly maintenance treatment along with weekly epirubicin treatment, or a combined treatment protocol along with this, was statistically more effective than weekly epirubicin treatment protocol alone ($0.01 < p < 0.05$).

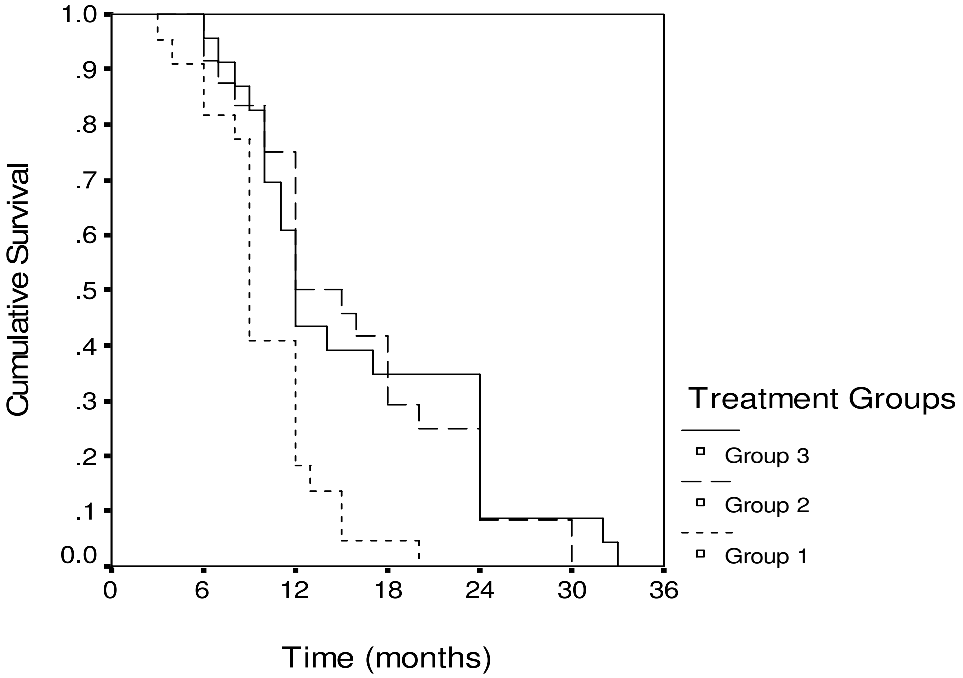

**FIGURE 7.** Kaplan-Meier survival curve of the patients in three treatment groups with respect to months.

In the three groups, no complications that would require the discontinuance of treatment were seen (Table 5). Leukopenia was grade 1-2 in two patients in both groups. However, blood transfusions were made due to the anemia that developed in all three groups. It was observed that the anemia generally developed after the fourth dose. In the first group in four patients and in other groups in one patient, the treatment was suspended for 3 weeks due to thrombocytopenia. Also, in two patients in the three groups, visible pigmentation developed in the fingernails. In three patients in each of the first and second groups, although nausea/vomiting of grade 1-2 was seen, grade 3-4 nausea/vomiting was seen in eight patients in third group when the estramustine dose was administered, and medical treatment was required. In two patients, these complaints diminished in time, yet six patients used the pharmaceutical intermittently due to this reason. In the first two groups, there were no subjective findings, pointing to a cardiotoxic effect. However, in two of our patients in the third group, thromboembolic complication was seen and these patients were lost at the $10^{th}$ and $12^{th}$ months of treatment.

## DISCUSSION

The therapeutic dose of epirubicin, used in the treatment of HRPC patients, has not been stated precisely. In addition, due to the cardiotoxic effect of epirubicin, along with its myelosuppressive effect, lifetime cumulative dose is restrictive.

**TABLE 5**
**Toxicity of Chemotherapy in the Treatment Groups According to WHO Criteria**

| | Group 1 (n = 22) No. (%) | Group 2 (n = 24) No. (%) | Group 3 (n = 23) No. (%) |
|---|---|---|---|
| Leukopenia* | | | |
| WHO grade 1-2 | 2 (9%) | 2 (8%) | 2 (9%) |
| WHO grade 3-4 | — | — | — |
| Anemia | | | |
| WHO grade 1-2 | 3 (14%) | 2 (8%) | 2 (9%) |
| WHO grade 3-4 | 1 (5%) | 2 (8%) | 2 (9%) |
| Thrombocytopenia | | | |
| WHO grade 1-2 | 4 (18%) | 1 (4%) | 1 (4%) |
| WHO grade 3-4 | — | — | — |
| Diarrhea | | | |
| WHO grade 1-2 | — | 1 (4%) | 2 (9%) |
| WHO grade 3-4 | — | — | — |
| Nausea/vomiting | | | |
| WHO grade 1-2 | 3 (14%) | 2 (8%) | 3 (13%) |
| WHO grade 3-4 | — | — | 8 (35%) |
| Alopecia | | | |
| WHO grade 1-2 | 8 (36%) | 11 (46%) | 9 (39%) |
| WHO grade 3-4 | — | — | — |
| Cardiotoxicity | | | |
| WHO grade 1-2 | — | — | — |
| WHO grade 3-4 | — | — | 2 (9%) |
| Gynecomastia | | | |
| WHO grade 1-2 | — | — | 4 (17%) |
| WHO grade 3-4 | — | — | — |

Just like all cytostatic pharmaceuticals, objective and subjective response rates differ according to the used dose and duration. Neri et al.[14] obtained a 28% partial response in a 16-week treatment with a 35 $mg/m^2$/week dose. Francini et al.[6] applied an epirubicin treatment for 12 weeks at a 30 $mg/m^2$/week dose and reported a life span of 12.5 months. Elomaa et al.[15] provided a 69% subjective response and a life span of 15 months at 25 $mg/m^2$/week doses.

Different results have been obtained in high-dose epirubicin studies. Tannock et al.[16] used epirubicin every 3 weeks at 75 $mg/m^2$ and provided a partial response of 12%. However, since they observed that the myelosuppressive effect at these doses was great, they did not recommend them. However, Delaere et al.[1] provided a 38% objective response to the monthly treatment application with 90 $mg/m^2$ doses, and recommended their protocols since they did not see any serious myelosuppressive or cardiotoxic side effects.

In the National Prostatic Cancer Project studies, a success rate of 0–35% in treatment protocols was reported, made by using EMP along with epirubicin[3]. Hernes et al.[17] reported that no objective responses were obtained with high-dose epirubicin treatment along with EMP, and the average life span was 6 months. Different results have been obtained from the combinations of EMP with vinblastine, etoposide, or docetaxel, other than epirubicin. Hudes et al.[18] reported that a 40% response was obtained from the EMP-vinblastine combination. Pienta et al.[19] reported a 50% objective response in combination with etoposide. Petryak et al.[20] reported more than a 50% decrease in PSA in 63% of the patients and 28% objective responses in an EMP combination with docetaxel. Also, Petryak reported a 20-month life span using a docetaxel and EMP combined treatment in a phase 2 study[21]. Similarly

Savarese et al.[22] used hydrocortisone in addition to docetaxel and EMP, and observed a 68% PSA response and 50% objective response. Similarly, in a study made with paclitaxel and EMP, a 22% objective response and 17-month life span was observed[23]. In a meta-analysis study performed with EMP and using various chemotherapy agents, it was shown that the combined treatment protocol was superior with respect to PSA response time and life span[24].

In our study, in accordance with the literature, a life span of 10.3 months was obtained in patients who were given only weekly doses. However, in two groups that received monthly maintenance treatment with weekly dose and combined treatment with EMP, life spans of 15.8 and 16.5 months, respectively, were provided. Also, in the first group, although an increase was seen in subjective and objective findings 4 months after the commencement of the treatment, an increase in subjective and objective findings was seen in the other two groups in the 6$^{th}$ and 8.4$^{th}$ months. In group 1 (induction epirubicin course of 8 weeks) and group 2 (maintenance epirubicin), the pain score showed a significant decrease at the 3$^{rd}$ and 6$^{th}$ months of follow-up when there was a significant increase at the 9$^{th}$ month. On the other hand, a statistically significant decrease was observed in group 3 (combined therapy) at the 9$^{th}$ month. In PSA levels, a statistically significant increase was observed at the 6$^{th}$ month in group 1, but in other groups, in contrary, a decrease at the 9$^{th}$ month, even if it was nonsignificant, was seen. These results were evaluated as that the maintenance therapy and combined therapy were both effective on PSA levels long term, but as subjective findings were much improved for a long time period in the combined therapy group, it was thought that this group was more effective.

In all three treatment groups, myelosuppressive effects were seen, yet these were not powerful enough for the discontinuance of treatment. Since cumulative dose was not exceeded, cardiotoxic effects connected to the use of epirubicin were not observed. However, the nausea/vomiting effect of EMP used in third treatment group affected the continuity of the treatment. Also, death due to thromboembolism was seen in connection with EMP.

The small number of patients in all three treatment protocols is not sufficient to emphasize the effectiveness and complications of the treatment. In any case, it can be seen that the second treatment protocol, in which maintenance treatment was administered for HRPC, is superior to the first group, with respect to treatment response time and life span. Similarly, although the combined treatment group is accepted as more effective than the first group, it is seen that EMP deteriorated the quality of life due to toxic effects. Because of this reason, the usage of a combined treatment protocol may be seen as unfavorable, yet in the literature, it is seen that the objective response to the combined treatments with EMP was high. Because of this reason, when usage is considered according to its effectiveness in treatment, medical measures must be adopted for known side effects.
